# A new spectral invariant for quantum graphs

**DOI:** 10.1038/s41598-021-94331-0

**Published:** 2021-07-28

**Authors:** Michał Ławniczak, Pavel Kurasov, Szymon Bauch, Małgorzata Białous, Afshin Akhshani, Leszek Sirko

**Affiliations:** 1 Institute of Physics, Polish Academy of Sciences, Aleja Lotników 32/46, 02-668 Warszawa, Poland; 2 Department of Mathematics, Stockholm University, 106 91 Stockholm, Sweden

**Keywords:** Physics, Quantum physics, Quantum mechanics

## Abstract

The Euler characteristic i.e., the difference between the number of vertices |*V*| and edges |*E*| is the most important topological characteristic of a graph. However, to describe spectral properties of differential equations with mixed Dirichlet and Neumann vertex conditions it is necessary to introduce a new spectral invariant, the generalized Euler characteristic $\chi _G:= |V|-|V_D|-|E|$, with $|V_D|$ denoting the number of Dirichlet vertices. We demonstrate theoretically and experimentally that the generalized Euler characteristic $\chi _G$ of quantum graphs and microwave networks can be determined from small sets of lowest eigenfrequencies. If the topology of the graph is known, the generalized Euler characteristic $\chi _G$ can be used to determine the number of Dirichlet vertices. That makes the generalized Euler characteristic $\chi _G$ a new powerful tool for studying of physical systems modeled by differential equations on metric graphs including isoscattering and neural networks where both Neumann and Dirichlet boundary conditions occur.

## Introduction

The problem of seven bridges of Königsberg considered by Leonhard Euler in 1736^1^ laid the foundation of classical, combinatorial graph theory and topology. Two hundred years later Linus Pauling^2^ applied the concept of graphs to describe the motion of a quantum particle in a physical network. This approach, now known as the quantum graph model, is widely used in the study of physical systems, e.g., quantum wires^3^, mesoscopic quantum system^4,5^, spectra of graphene and carbon nanotubes^6^, Bose–Einstein condensates^7,8^, Anderson localization^9^ and optical wave guides^10^. In 1948, Feynman^11^ introduced diagrams (graphs) as pictorial representation of the mathematical expressions describing the behavior and interaction of subatomic particles.

The theory of quantum graphs has been a subject of intense research^12–17^. The metric graph $\Gamma =(V,E)$ consists of edges $e \in E$ being intervals of the length $l_e$ on the real line $\mathbb {R}$ connected at the vertices $v \in V$, which are defined as the unions of edges endpoints. Such a graph uniquely determines the Laplace operator $L(\Gamma ) = -\frac{d^2}{dx^2}$ acting in the Hilbert space of square integrable functions. $L(\Gamma )$ is self-adjoint, its spectrum is discrete and nonnegative^15^. When a graph has only vertices $V_{N}$ with Neumann (called also standard, natural) vertex boundary conditions: functions are continuous at vertices and the sums of their oriented derivatives at vertices are equal zero, then the Laplacian has a simple zero eigenvalue with the eigenfunction being a constant. Introducing even one vertex $V_D$ with the Dirichlet boundary condition (a functions is zero at the vertex) into the graph causes that spectral multiplicity of the eigenvalue 0 to become zero instead of one.

In this article we generalize the notion of the Euler characteristic^18^ to graphs possessing vertices with both Neumann and Dirichlet boundary conditions. We show that for such graphs it is possible to determine the generalized Euler characteristic $\chi _G$ from small sets of their lowest eigenvalues $\lambda _1, \ldots , \lambda _N$.

The experimental verification of our theoretical findings is carried out using the spectra of microwave networks which simulate quantum graphs^19–24^. This is attainable because the one-dimensional Schrödinger equation describing quantum graphs is formally equivalent to the telegrapher’s equation for microwave networks^19,22^. The microwave networks are extremely useful in studying quantum and wave chaos. Uniquely they allow for the experimental realization of systems described by the main three symmetry classes in random-matrix theory (RMT): systems with preserved time reversal symmetry (TRS) represented by Gaussian orthogonal ensemble (GOE)^18–21,23,25,26^; systems with preserved TRS and half-spin represented by Gaussian symplectic ensemble (GSE)^27,28^; systems without TRS represented by Gaussian unitary ensemble (GUE)^19,24,29–33^. The chiral orthogonal, unitary, and symplectic ensembles^34^ have been recently realized using microwave networks. Microwave networks have been also used to study a topological edge invariant^35,36^ and the photon number statistics of coherent light^37^. Therefore, microwave networks as well as flat microwave cavities^38–47^ and Rydberg atoms strongly driven by microwave fields^48–51^ have become one of the most important model systems, that are successfully used in experimental modeling of complex quantum systems.

## The generalized Euler characteristic for quantum graphs with Dirichlet boundary conditions

One of the most important characteristic of a metric graph is the Euler characteristic

1 $$\begin{aligned} \chi =|V|-|E|, \end{aligned}$$

where |*V*| and |*E*| denote the number of vertices and edges, respectively.

The Euler characteristic $\chi$ determines another important quantity characterizing the graph, the number of independent cycles $\beta$ in it

2 $$\begin{aligned} \beta = |E|-|V|+1 \equiv 1-\chi . \end{aligned}$$

This number, known also as the first Betti number, tells us how many edges have to be removed from the connected graph in order to turn it into a tree graph.

In this article we will demonstrate that vertices with Dirichlet boundary conditions (Dirichlet vertices) play an important role in graph spectral characteristics, leading to a new spectral invariant, called generalized Euler characteristic

3 $$\begin{aligned} \chi _G = \chi - |V_D|, \end{aligned}$$

where $|V_D|$ is the number of Dirichlet vertices.

Our goal is to relate the generalized Euler characteristic $\chi _G$ to the spectrum of the Laplace operator $L = - \frac{d^2}{dx^2}$ on the metric graph $\Gamma$. One usually assumes standard vertex conditions: continuity of functions and Neumann conditions on the sum of the first derivatives. This case was comprehensively treated in Ref.^18^. Here, we will generalize the results obtained in Ref.^18^ to the case of mixed standard (Neumann) and Dirichlet vertex conditions. We assume that Dirichlet conditions are imposed only at degree one vertices of pendent edges since higher degree Dirichlet vertices should be treated as separate degree one Dirichlet vertices. Standard vertex conditions at degree one vertices are equivalent to Neumann conditions since the continuity condition is redundant. In what follows, the degree one vertices in $\Gamma$ are divided into two classes: Neumann and Dirichlet vertices, respectively. We shall always assume standard conditions at the vertices with the degree larger than 1.

The Laplace operator is self-adjoint and is uniquely determined by $\Gamma$ and the set $V_D$ of Dirichlet vertices. The spectrum is discrete bounded from below $0<\lambda _1< \lambda _2 \le \lambda _3 < \dots$ and satisfies Weyl’s law

4 $$\begin{aligned} \lambda _n = \Big (\frac{\pi }{\mathcal {L}}\Big )^2n^2 + \mathcal {O}(n) \,, \end{aligned}$$

where $\mathcal {L}$ is the total length of the graph and $\mathcal {O}(n)$ is a function which divided by *n* in the limit $n \rightarrow \infty$ is bounded by a constant. Note that $\lambda _1 \ne 0$ provided $\Gamma$ is connected and $|V_D|>0$ holds.

For graphs with standard vertex conditions at all vertices the following formula for the Euler characteristic was proven^18^:

5 $$ \chi = 2 + 8 \pi ^2\sum _{\begin{array}{c} k_n \in \Sigma (L^\text{st} (\Gamma )) \\ k_n \ne 0 \end{array}} \frac{\sin (k_n/t)}{(k_n/t) \big ( (2\pi )^2 - (k_n/t)^2 \big )} \vert _{t \ge t_0}, $$

where $\Sigma (L^\text{st} (\Gamma ))$ denotes the spectrum of the Laplacian $L^\text{st} (\Gamma )$ with standard vertex conditions, taken in the square root scale, i.e., the numbers $k_n$ are the square roots of the eigenenergies $\lambda _n$ and $t_{0} = \frac{1}{2l_{min}}$, where $l_{min}$ is the length of the shortest edge of the graph. If the summation sequence in Eq. (5) contains an infinite number of terms, $t \ge t_0$ is an arbitrary free parameter. However, we will show that in the case of limited number of eigenvalues $k_n$ the value of *t* should be limited from above by some $t_{max}$.

Formula (5) was obtained using the trace formula connecting the spectrum of the Laplacian to the set of periodic orbits on the metric graph^52–55^ applying it to a carefully chosen test function^18,56–58^. Our objective is to generalize this formula by including Dirichlet vertices. This is important because for example both Dirichlet and Neumann conditions appear in the isoscattering and neural networks, where for the latter ones they appear naturally as a result of learning procedures^59^.

Let $$ \Gamma $$ be a finite connected metric graph with $$ |V_D |$$ Dirichlet vertices $$ v_1, v_2, \dots , v_{|V_D|} $$. We assume standard vertex conditions at all other vertices. The corresponding Laplace operator will be denoted by $$ L^\text{st,D} (\Gamma )$$. Let us double the graph by adding to $$ \Gamma $$ another copy of the same graph and gluing them by joining pairwise the vertices $$ v_j, \; j = 1,2, \dots , |V_D|. $$ Let us denote the metric graph obtained in this way by $$ \Gamma _2. $$ This graph is symmetric with respect to the exchange of the respective points on the two copies of $$ \Gamma $$. Hence all eigenfunctions and the spectrum can be divided into two classes:

- symmetric eigenfunctions satisfying Neumann conditions at $v_j, j=1,2,\dots , |V_D|$, the spectrum coincides with the spectrum of the standard Laplacian $L^\text{st} (\Gamma )$;
- antisymmetric eigenfunctions satisfying Dirichlet conditions at $v_j, j=1,2,\dots , |V_D|$, the spectrum coincides with the spectrum of $L^\text{st,D} (\Gamma )$ for which $k_n \ne 0$.

Let $\chi$ be the Euler characteristic of $\Gamma$, then $\Gamma _2$ has $-2\chi + |V_D|-1$ independent cycles and its Euler characteristic is $2 \chi - |V_D|$ .

Applying the formula (5) to standard Laplacians on $\Gamma$ and $\Gamma _2$ we get

6 $$\begin{aligned} 2 \chi - |V_D|&= 2 + 8 \pi ^2\ \sum _{ \begin{array}{c} k_n \in \Sigma (L^\text{st} (\Gamma _2)) \\ k_n \ne 0 \end{array} } \frac{\sin (k_n/t)}{(k_n/t) \big ( (2\pi )^2 - (k_n/t)^2 \big )} \\&= \underbrace{2 + 8 \pi ^2\sum _{ \begin{array}{c} k_n \in \Sigma (L^\text{st} (\Gamma )) \\ k_n \ne 0 \end{array}} \frac{\sin (k_n/t)}{(k_n/t) \big ( (2\pi )^2 - (k_n/t)^2 \big )}}_{\displaystyle = \chi } + 8 \pi ^2\sum _{ k_n \in \Sigma (L^\text{st,D} (\Gamma ))} \frac{\sin (k_n/t)}{(k_n/t) \big ( (2\pi )^2 - (k_n/t)^2 \big )}, \end{aligned} $$

where $\Sigma ( \cdot )$ denote the spectra of different Laplacians on $\Gamma _2$ and $\Gamma$, again considered in the square root scale. Then the formula (5) implies that

7 $$ \chi _{G} : = \chi  - |V_{D} | = 8\pi ^{2} \sum\limits_{{k_{n}  \in \Sigma (L^{{{\text{st}},{\text{D}}}} (\Gamma ))}} {\frac{{\sin (k_{n} /t)}}{{(k_{n} /t)((2\pi )^{2}  - (k_{n} /t)^{2} )}}} . $$

Hence the Euler characteristic $\chi$ alone is not a proper spectral invariant in the case of Laplacians with Dirichlet vertices, we have to replace it by the invariant $\chi _G$ introduced above. It generalizes naturally the Euler characteristic and has the following important property: any two isospectral graphs with mixed standard and Dirichlet conditions necessarily have the same $\chi _G$. This property of $\chi _G$ will be checked experimentally in this article using two isoscattering and therefore isospectral microwave networks^21,60^.

It is important to point out that the generalized Euler characteristic $\chi _G$ is an integer. Therefore, to determine its value precisely it is enough to calculate it with an accuracy better than 1/2. It means that infinite series on the right-hand of the Eq. (7) can be substituted with a finite sum. This is essential because in the real world of physical measurements it is not possible to determine the entire spectrum of the tested system. In microwave experiments internal absorption and openness of systems limit from the top the frequency range in which eigenfrequencies (resonances) can be determined.

To determine how many terms (resonances) in the formula (7) are required to get the value of $\chi _G$ with the accuracy $\epsilon$ better than 1/2 the following relations will be considered assuming $t \ge t_0$:

8 $$\begin{aligned} \begin{array}{ccl} \displaystyle X_K^{G}(t) &{} := &{} \displaystyle {8\pi ^{2}}\sum _{n=1}^{K}\frac{\sin (k_{n}/t)}{(k_{n}/t)((2\pi )^{2} - (k_{n}/t)^{2})} \\ \displaystyle \epsilon &{} := &{} \displaystyle \Big | \underbrace{X^{G}(t) }_{\displaystyle = \chi _G}-X_K^{G}(t) \Big | = \Big |{8\pi ^{2}}\sum _{n=K+1}^{\infty }\frac{\sin (k_{n}/t)}{(k_{n}/t)((2\pi )^{2} - (k_{n}/t)^{2})} \Big |. \end{array} \end{aligned}$$

It is necessary to choose *K* sufficiently large to guarantee $\epsilon < 1/2$. Such an estimate for $t=t_0=\frac{1}{2l_{min}}$ has been carried out in Appendix of Ref.^18^ in the case of standard boundary conditions. The existence of Dirichlet vertices does not change the estimate for the minimum number of resonances:

9 $$\begin{aligned} K \ge \left\lfloor {|V| + 2\mathcal L t \left[ 1-\exp \left( \frac{-\epsilon \pi }{\mathcal L t}\right) \right] ^{-1/2}}\right\rfloor , \end{aligned}$$

where |*V*| is the total number of graph vertices. The smallest number of resonances $K=K_{min}$ is obtained by substituting the smallest allowed value of *t*, that is $t=t_0$. In the calculation of $K_{min}$ we will also assume that $\epsilon =1/4$.

Equation (9) demonstrates that in the evaluation of $$X_K^{G}(t=t_0)$$ (Eq. (8)) it is enough to use only a limited number of terms $$K=K_{min}$$. In such a case, as mentioned above, in the behavior of $$ X_{K_{min}}^{G}(t)$$ for $$t>t_0$$ one expects to observe a plateau which will be destroyed for $$t \simeq t_{max}$$. We are interested in getting a rough estimate for the maximum allowed value of *t*. For this purpose for $$\mathcal L t \gg 1$$ we will approximate (9) with a formula

10 $$\begin{aligned} K \ge \left\lfloor { |V| + \frac{2}{\sqrt{\epsilon \pi }}(\mathcal L t)^{3/2} }\right\rfloor . \end{aligned}$$

Assuming that $\epsilon =1/4$ and $t=t_0$, for which $K=K_{min}$, Eq.(10) can be used to define the maximum allowed value of $t_{max}$ for which $K=K_{min}$ is preserved but $\epsilon$ was increased to $\epsilon _{max}=1$. This yields a simple relationship between an approximated $t_{max}$ and $t_0$

11 $$\begin{aligned} t_{max} \simeq 4^{1/3}t_0 \simeq 1.59t_0. \end{aligned}$$

## How to hear the boundary conditions of quantum graphs?

From the theoretical point of view looking at the eigenvalue $\lambda = 0$ one can easily identify whether some of the vertex conditions on a connected metric graph are Dirichlet or not: $\lambda = 0$ is an eigenvalue of the Laplacian if and only if all vertex conditions are Neumann.

To determine the number of Dirichlet vertices one may use

- the generalized Euler characteristic $\chi _G = \chi - |V_D|$ determined by the Laplacian spectrum via explicit formula (7);
- the conventional Euler characteristic $\chi = |V|-|E|$, which can be obtained, e.g., by visually examining the number $\beta = 1- \chi$ of independent cycles in the graph.

Having determined these numbers, the number of Dirichlet vertices is given by

12 $$\begin{aligned} |V_D|=\chi -\chi _G. \end{aligned}$$

This formula reminds that the generalized Euler characteristic is an integer that cannot exceed the topological Euler characteristic $\chi$.

From the experimental point of view the situation is more complicated because identification whether $\lambda = 0$ is an eigenvalue of an investigated system maybe impossible. In such a case, the number of Dirichlet vertices can be evaluated in the following way.

Taking into account the properties of the formula (5) one can find out that if the generalized Euler characteristic $\chi _G$ evaluated from the spectral formula (7) (right-hand side of Eq. (7)) fulfills the condition

13 $$\begin{aligned} \chi -\chi _G \ne 2 \end{aligned}$$

one deals with a graph which possesses Dirichlet vertices and their number is given by the formula (12).

In the case when

14 $$\begin{aligned} \chi - 8 \pi ^2\sum _{ \begin{array}{c} k_n \in \Sigma \\ k_n \ne 0 \end{array}} \frac{\sin (k_n/t)}{(k_n/t) \big ( (2\pi )^2 - (k_n/t)^2 \big )} = 2 \end{aligned}$$

one deals with a graph which possesses either 0 or 2 Dirichlet vertices and an additional information is required to find their actual number.

The latter case can be illustrated using a single interval graph ($|V|=2$, $|E|=1$, $\chi =1$) of length $\pi$. The spectrum of the standard Laplacian is $0, 1, 2, \dots$, while the spectrum of the Dirichlet Laplacian, with two Dirichlet vertices, $|V_D|=2$, is $1,2,3 \dots$. Summing the series (5) and (7) over non-zero spectra of the respective Laplacians one obtains $\chi -2=-1$ and $\chi _G=-1$. The same outcome of the calculations shows that we cannot determine whether we deal with the standard Laplacian, where we lost $\lambda =0$ eigenvalue, or with the Dirichlet Laplacian, where such an eigenvalue does not exist.

However, if the experiment yields that $\lambda = 0$ is not an eigenvalue of the system, the number of Dirichlet vertices is directly given by the formula (12).

## Experimental setup and methodology of measurements

To test the formula (7) for the generalized Euler characteristic $\chi _{G}$ we identified experimentally the required by Eq. (9) numbers of resonances of microwave networks simulating quantum graphs without loops with Dirichlet boundary conditions.

**Figure 1:**
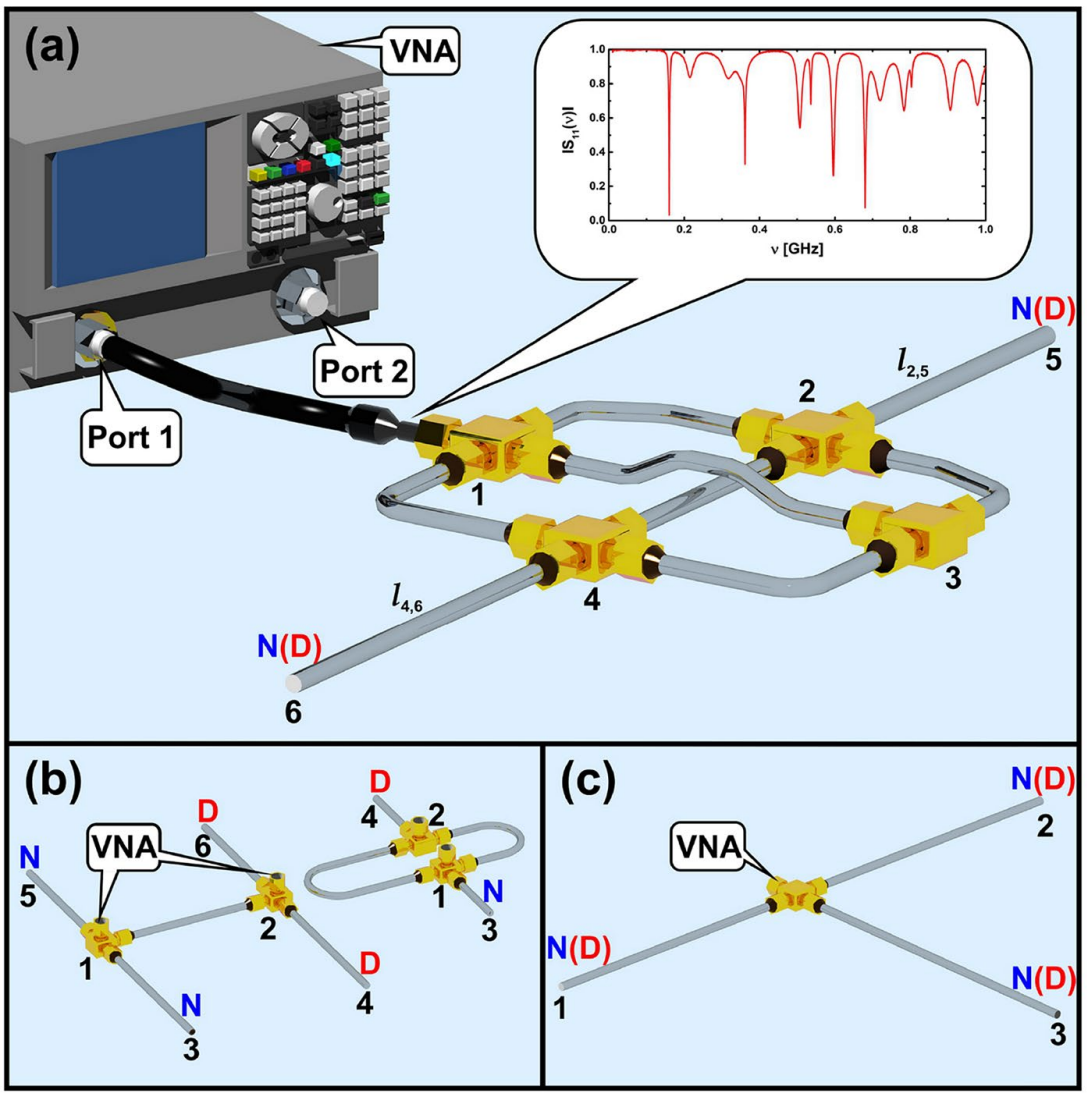
(**a**) The experimental setup consists of the Agilent E8364B vector network analyzer (VNA) and the HP 85133-616 flexible microwave cable that connects the VNA with the measured network simulating quantum graphs $\Gamma (6,8,|V_D|=0,1,2)$ possessing two tail-like edges $l_{2,5}$ and $l_{4,6}$. The resonances of the network were measured for various boundary conditions of the tail-like edges $l_{2,5}$ and $l_{4,6}$ marked with *N* and *D* capital letters for Neumann and Dirichlet boundary conditions, respectively. The same convention will be also used throughout the other panels. The inset shows an example of the modulus of a single port scattering matrix $|S_{11}(\nu )|$ of the network $\Gamma (6,8,|V_D|=2)$ measured in a frequency range 0.001–1 GHz. (**b**) The isoscattering networks simulating quantum graphs $\Gamma (4,4,1)$ (O-graph) and $\Gamma (6,5,2)$ (H-graph). In this case the two-port scattering matrices $\hat{S}_O(\nu )$ and $\hat{S}_H(\nu )$ were measured in a function of microwave frequency $\nu$^21,60^. (**c**) The microwave network which was used to simulate the star graphs $\Gamma (4,3,0)$, $\Gamma (4,3,1)$, $\Gamma (4,3,2)$ and $\Gamma (4,3,3)$. The network consists one vertex of valency four and three edges terminated with the vertices of valency one.

The experimental setup (see Fig. 1a), standard for such measurements, consists of the Agilent E8364B vector network analyzer (VNA) and the HP 85133-616 high class flexible microwave cable that connects the VNA with the measured network. Such a cable is equivalent to attaching an infinite lead to a quantum graph^18,25^. To eliminate the influence of the external to the network elements on measurement results the VNA was calibrated with an Agilent 4691-60004 electronic calibration module.

Quantum graphs are simulated by microwave networks containing coaxial cables and junctions that correspond to the edges and vertices of the graphs. The cables are composed of an inner conductor of a radius $r_{1} = 0.05$ cm surrounded by the dielectric material (Teflon) and an outer concentric conductor with an inner radius $r_{2} = 0.15$ cm. The dielectric constant of Teflon measured by us equals $\varepsilon =2.06$. So the cut-off frequency of the TE$_{11}$ below which only the fundamental TEM can propagate in the cable^61,62^ is $\upsilon _{cut}=\frac{c}{\pi (r_{1}+r_{2})\sqrt{\varepsilon }}=33$ GHz. The physical lengths $l_{ph}$ of the cables determine the optical lengths of the graph edges through relationship $l_{opt}= \sqrt{\varepsilon }l_{ph}$.

In order to identify the resonances of the networks in a required frequency range, starting from the lowest one $\nu _1$, we carried out the measurements of their one-port scattering matrix $S_{11}(\nu )$. To verify the completeness of the sets of resonances the fluctuating part of the integrated spectral counting function $N_{fl}(\nu _{i}) = N(\nu _{i}) - N_{av}(\nu _{i})$, that is the difference of the number of eigenfrequencies $N(\nu _{i}) = i$ for ordered frequencies $\nu _{1} \le \nu _{2} \le \cdots$ and the average number of eigenfrequencies $N_{av}(\nu _{i})$ calculated for the tested frequency range, was analyzed. The resonance frequencies give directly the real part of the wave vectors Re $k_n = \frac{2\pi }{c}\nu _{n}$. In the case of isoscattering networks the two-port scattering matrix $\hat{S}(\nu )$ was measured and the resonances were identified from the amplitude of the determinant of the scattering matrix $\hat{S}(\nu )$. The details of this experimental procedure are given in details in Refs.^21,60^.

## Experimental results

To simplify the description of the networks we introduce the following notation of graphs and networks $\Gamma (|V|,|E|,|V_D|)$, where $|V|=|V_N|+|V_D|$. A network $\Gamma (|V|,|E|,|V_D|)$ contains |*V*| vertices, including $|V_N|$ and $|V_D|$ vertices with Neumann and Dirichlet boundary conditions and |*E*| edges.

In order to test the formula (7) we considered the microwave networks simulating the following quantum graphs: 6-vertex graphs with two tail-like edges $\Gamma (6,8,|V_D|=0,1,2)$, two isoscattering graphs $\Gamma (4,4,1)$ (O-graph) and $\Gamma (6,5,2)$ (H-graph), and the star graphs $\Gamma (4,3,|V_D|=0,1,2,3)$.

**Figure 2:**
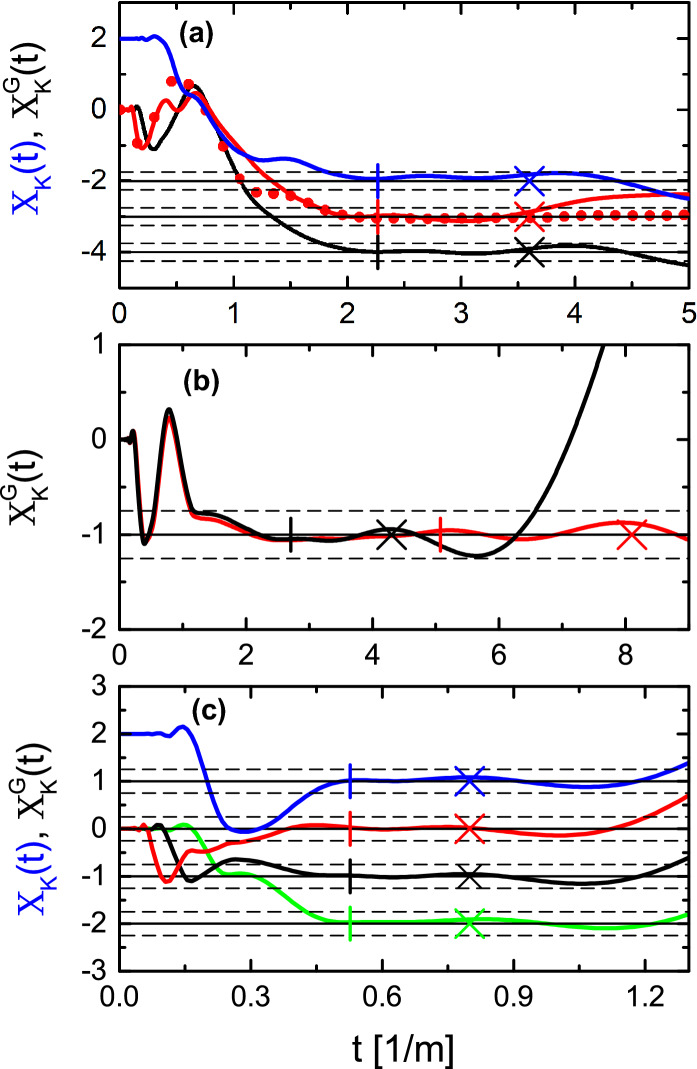
The approximation functions for the Euler characteristic $X_K(t)$ and the generalized Euler characteristic $X_K^G(t)$ evaluated for different networks in a function of the parameter *t*. (**a**) The approximation function $X_K(t)$ (blue full line) calculated for the 6-vertex network $\Gamma (6,8,0)$ with two tail-like edges with Neumann boundary conditions. The approximation functions $X_K^G(t)$ evaluated for the networks $\Gamma (6,8,1)$ (red full line—Dirichlet vertex on the edge $l_{2,5}$; red dotted line—Dirichlet vertex on the edge $l_{4,6}$) and $\Gamma (6,8,2)$ (black full line), respectively. (**b**) The approximation functions $X_K^G(t)$ for the isoscattering networks $\Gamma (4,4,1)$ (O-graph; red full line) and $\Gamma (6,5,2)$ (H-graph; black full line). (**c**) The approximation functions $X_K(t)$ evaluated for the star network $\Gamma (4,3,0)$ (full blue line) and $X_K^G(t)$ for the star networks $\Gamma (4,3,1)$ (red full line), $\Gamma (4,3,2)$ (black full line) and $\Gamma (4,3,3)$ (green full line), respectively. The vertical lines show the values of $t_0$ for the analyzed networks. The crosses denote approximated values of $t_{max}$ limiting from above the plateaux in $X_K(t)$ and $X_K^G(t)$. The black broken lines show the limits of the expected errors $\chi \pm 1/4$ or $\chi _G \pm 1/4$.

### Microwave networks $\Gamma (6,8,|V_D|=0,1,2)$

The network simulating quantum graphs $\Gamma (6,8,|V_D|=0,1,2)$ (see Fig. 1a) of the total optical length $\mathcal {L} = 2.377$ m consists of six vertices and eight edges of the lengths $l_{1,2}= 0.237$ m, $l_{1,3}= 0.289$ m, $l_{1,4}= 0.382$ m, $l_{2,3}= 0.261$ m, $l_{2,4}= l_{min} = 0.221$ m, $l_{3,4}= 0.249$ m, $l_{2,5} = 0.313$ m, $l_{4,6} = 0.425$ m. The lower indices are the numbers of the vertices connected by the edges. The minimum number of the resonances necessary to get the Euler characteristic with accuracy $\epsilon = 1/4$, $K_{min} = 35$, which corresponds to the frequency $\nu \simeq 2.25$ GHz. The values of the parameters $t_0=2.26$ and $t_{max} \simeq 3.6$. The resonances of the network were measured for various boundary conditions of the tail-like edges $l_{2,5}$ and $l_{4,6}$ denoted in Fig. 1a with *N* and *D* for Neumann and Dirichlet boundary conditions, respectively. Four combinations of the boundary conditions and the expected Euler characteristic $\chi$^18^ or generalized Euler characteristic $\chi _{G}$ are presented in Table 1.

**Table 1:** The Euler characteristic $\chi$ and the generalized Euler characteristic $\chi _{G}$ predicted for the microwave networks $\Gamma (6,8,|V_D|=0,1,2)$ for four combinations of Neumann (N) and Dirichlet (D) boundary conditions (BC) on the two tail-like edges $l_{2,5}$ and $l_{4,6}$.

$\text{ BC: } l_{2,5}$	$\text{ BC: } l_{4,6}$	$\;\;\; \chi \;\;\;$	$\;\; \chi _G \;\;$
*N*	*N*	$-2$	−
*N*	*D*	−	$-3$
*D*	*N*	−	$-3$
*D*	*D*	−	$-4$

The results obtained from the spectral formula (5) for the approximation function of the Euler characteristic $X_K(t)$ in Ref.^18^ and from Eq. (8) for the approximation function of the generalized Euler characteristic $X_K^G(t)$ as a function of *t* are shown in Fig. 2a. The approximation function $X_K(t)$ (blue full line) was calculated for the 6-vertex network $\Gamma (6,8,0)$ with two tail-like edges with Neumann boundary conditions. The approximation functions $X_K^G(t)$ were evaluated for the networks $\Gamma (6,8,1)$ (red full line—Dirichlet vertex (DV) on edge $l_{2,5}$, red dotted line—DV on edge $l_{4,6}$) and $\Gamma (6,8,2)$ (black full line), respectively. The Euler characteristic $\chi$ and the generalized Euler characteristic $\chi _G$ were obtained as the values of the plateaux observed in the approximation functions $X_K(t)$ and $X_K^G(t)$, respectively, for $t\ge t_{0}$, and $K = K_{min}$. The experimental values of the Euler characteristic $\chi$ and the generalized Euler characteristic $\chi _G$ are in agreement with the predicted theoretical ones shown in Table 1.

### Isoscattering microwave networks

The isoscattering microwave networks^21,26,60^ were investigated in order to extend a famous question of Mark Kac “Can one hear the shape of a drum?”, originally posed in the case of isospectral dissipationless systems, to the case of open graphs and networks. The isoscattering networks simulating quantum graphs $\Gamma (4,4,1)$ (O-graph), $\Gamma (6,5,2)$ (H-graph)^21,60^ (see Fig. 1b) were investigated in the frequency range 0.001–4.62 GHz. In this work we significantly extended our previous measurements of the two-port scattering matrices $\hat{S}_O(\nu )$ and $\hat{S}_H(\nu )$ of O- and H-networks, respectively, which in Ref.^60^ were reported only in the frequency range 0.001–3 GHz. The total optical length of the both networks is the same and amounts to $\mathcal {L} = 1.0504$ m. The network simulating the O-graph consists of four edges of the lengths $a=l_{min}=0.0985$ m, $2b=0.3694$ m, $a=l_{min}=0.0985$ m, $2c=0.4840$ m, and four vertices, one with the Dirichlet boundary condition terminating one of the edges *a*. The network simulating the H-graph consists of six edges $b=l_{min}=0.1847$ m, $c=0.2420$ m, $2a=0.1970$ m, $b=l_{min}=0.1847$ m, $c=0.2420$ m, and six vertices. Two of these vertices, terminating one edge of each pairs of *b* and *c*, have Dirichlet boundary conditions.

Since the isoscattering networks are also isospectral if it concerns their spectra identified from the amplitudes of the determinants of the scattering matrices $\hat{S}_O(\nu )$ and $\hat{S}_H(\nu )$, it is obvious from Eq. (7) that both networks should have the same generalized Euler characteristic $\chi _G$. The approximation function for the generalized Euler characteristic $X_K^G(t)$ in a function of *t* is presented in Fig. 2b for the O-network ($K_{min} = 32$, $t_0=5.08$, and $t_{max} \simeq 8.1$) and the H-network ($K_{min}=17$, $t_0=2.71$, and $t_{max} \simeq 4.3$) by red full and dotted lines, respectively. In both cases the approximation function $X_K^G(t)$ gives the same value $\chi _G=-1$ what should be expected in the case of the isoscattering networks.

Moreover, from the formal definition of the generalized Euler characteristic $\chi _G$ (see Eq. 3) we have

15 $$\begin{aligned} \chi _{G} = \left\{ \begin{array}{ll} 4-4-1 = -1, &{} \text{ for } \text{ O-graph }, \\ 6-5-2 = -1, &{} \text{ for } \text{ H-graph }, \end{array} \right. \end{aligned}$$

in full agreement with the experimental results.

### Star microwave networks

A star graph is a special type of a tree graph which contains at most one vertex of degree greater than one. Any quantum graph looks locally near vertices like a star graph therefore these simplest non-trivial graphs play a very important role in the graph theory^15^.

We examined the star graphs $\Gamma (4,3,0)$, $\Gamma (4,3,1)$, $\Gamma (4,3,2)$ and $\Gamma (4,3,3)$, with three edges, often called claws. The graphs were simulated by a network (see Fig. 1c) consisting of one vertex of valency four and three edges terminated with the vertices of valency one. The optical lengths of the edge are $l_{1} = l_{min}= 0.949$ m, $l_{2} = 1.115$ m and $l_{3} = 0.981$ m giving the total network length $\mathcal {L} = 3.045$ m. For $\epsilon =1/4$, $K_{min} = 9$, and $\nu _9 \simeq 0.47$ GHz. The values of the parameters: $t_0=0.53$ and $t_{max}\simeq 0.8$.

The Euler characteristic $\chi$^18^ of the graph $\Gamma (4,3,0)$ is $\chi = 4-3= 1$, since we deal with the graph without Dirichlet boundary conditions. In Fig. 2c, the approximation function for the Euler characteristic $X_K(t)$ (full blue line), which is close to 1, is shown as a function of *t* for the network $\Gamma (4,3,0)$. It is important to mention that the Euler characteristic for tree graphs possessing only Neumann vertices is always $\chi _{tree} = 1$ and is independent on the size of a tree graph.

If in a tree graph at least one vertex with the Dirichlet boundary condition is present one should use Eq. (7) to evaluate the generalized Euler characteristic $\chi _{G}$. For the star networks $\Gamma (4,3,1)$, $\Gamma (4,3,2)$ and $\Gamma (4,3,3)$ the generalized Euler characteristic $\chi _G$ is equal to 0, − 1, and − 2, respectively, which is clearly seen in the dependence of the approximation function for the generalized Euler characteristic $X_K^G(t)$ on the parameter *t* in Fig. 2c.

Using the star networks one can test in practice the procedure of identifying the number of Dirichlet boundary conditions in a quantum graph or microwave network. Microwave networks are so useful in simulation of quantum graphs because, additionally to the discussed earlier properties, their eigenvalue $\lambda =0$ can be also easily found by measuring the electric conductance $\mathcal {G}$. For microwave networks with standard boundary conditions, $\mathcal {G} = 0$, while for the networks with at least one Dirichlet boundary condition, $\mathcal {G} = +\infty$.

The direct measurements of the electric conductance yielded that the star networks $\Gamma (4,3,1)$, $\Gamma (4,3,2)$, and $\Gamma (4,3,3)$ possess at least one Dirichlet vertex. Therefore, using Eq. (12) one can easily found that the number of Dirichlet vertices in the above networks is, 1, 2, and 3, respectively, in agreement with the experimental realizations of the star graphs.

## Conclusions

We introduced a new spectral invariant: the generalized Euler characteristic $\chi _G=|V|-|E|-|V_D|$ of quantum graphs possessing standard (Neumann) and Dirichlet boundary conditions. We show theoretically and experimentally that the generalized Euler characteristic $\chi _G$ can be determined from small sets of the lowest eigenvalues $\lambda _1, \ldots , \lambda _N$ of graphs and microwave networks. We demonstrate that the generalized Euler characteristic $\chi _G$ together with the commonly known Euler characteristic $\chi =|V|-|E|$ can be applied to reveal (hear) the number of Dirichlet vertices in the investigated graphs and networks. The theoretical findings are illustrated and confirmed experimentally using microwave networks that showed that the generalized Euler characteristic $\chi _G$ is a new powerful tool for studying of quantum graphs and microwave networks, and as a consequence, all systems modeled by the equivalent differential equations.

## Data Availability

The data that support results presented in this paper and other findings of this study are available from the corresponding authors upon reasonable request.
